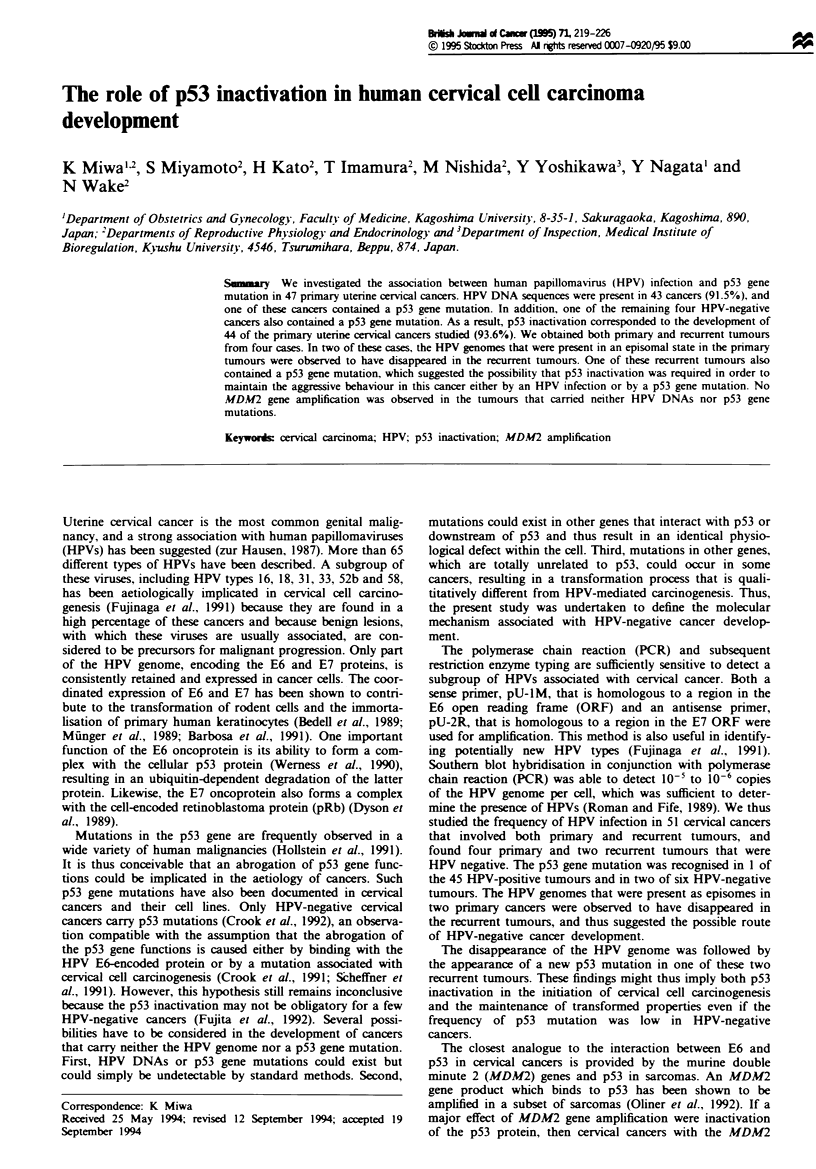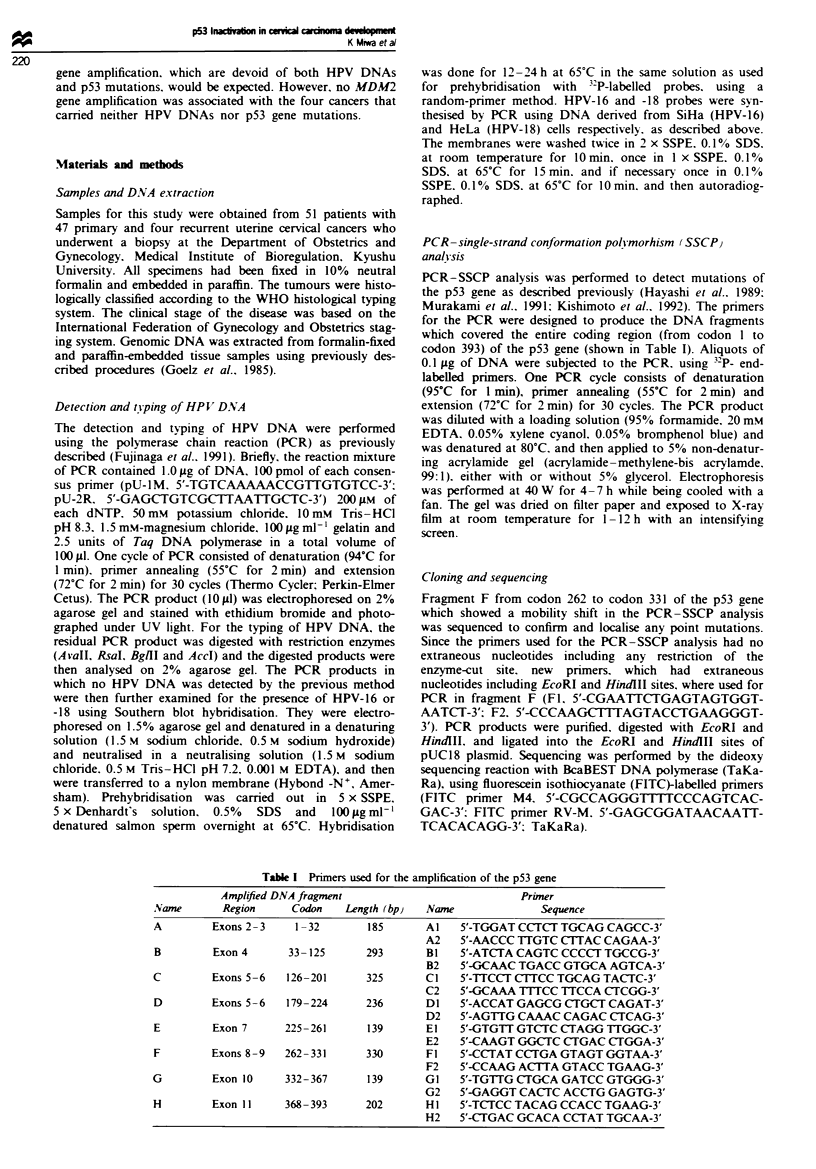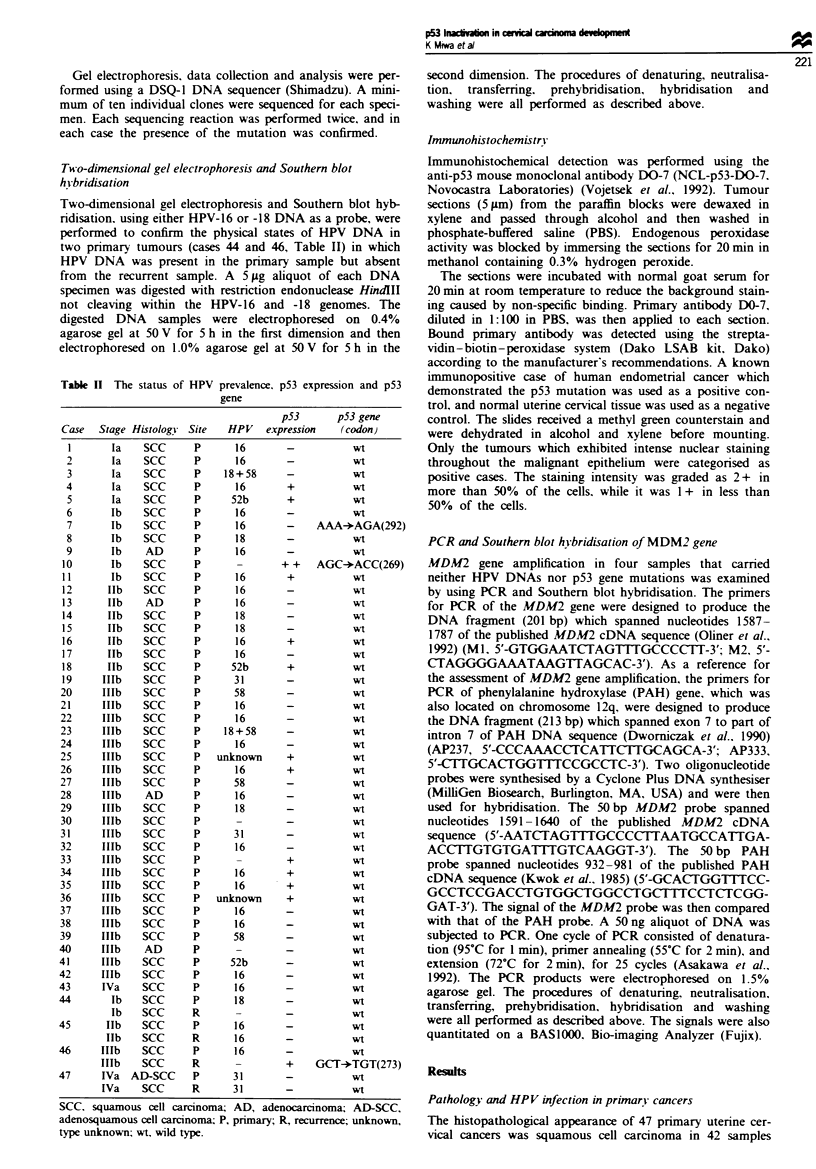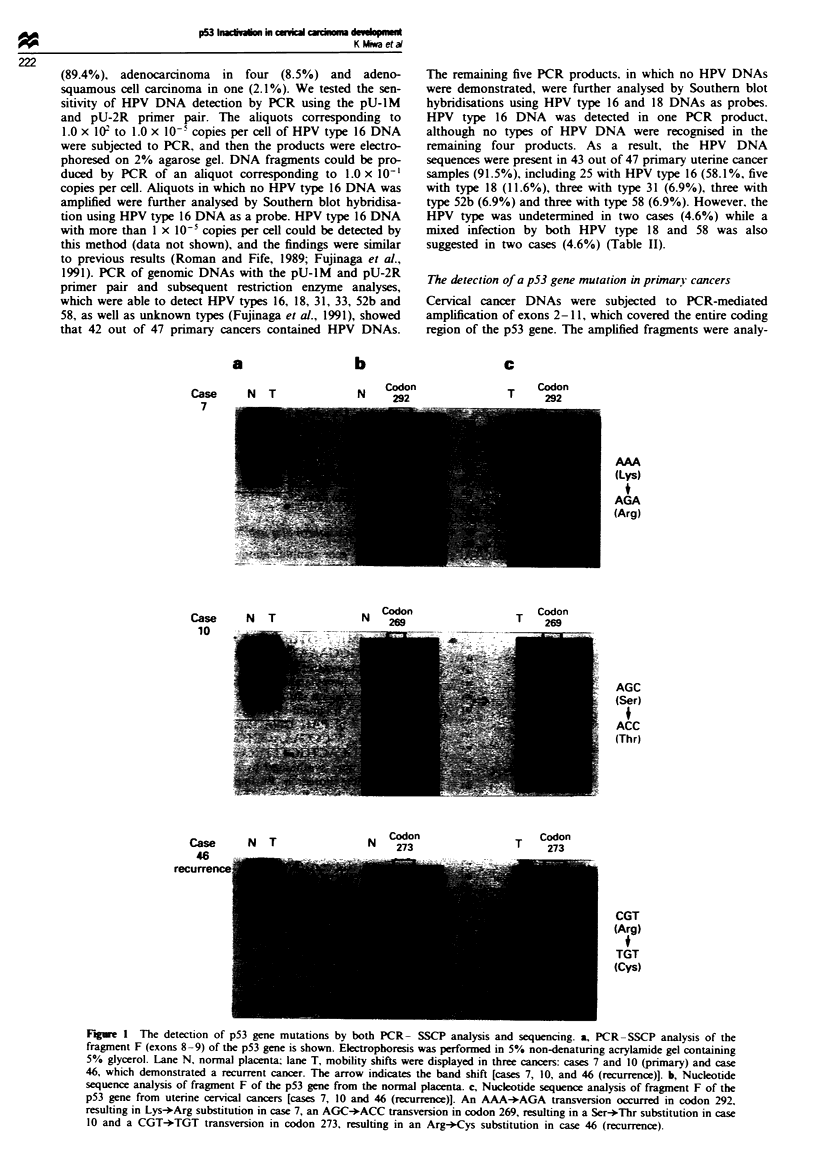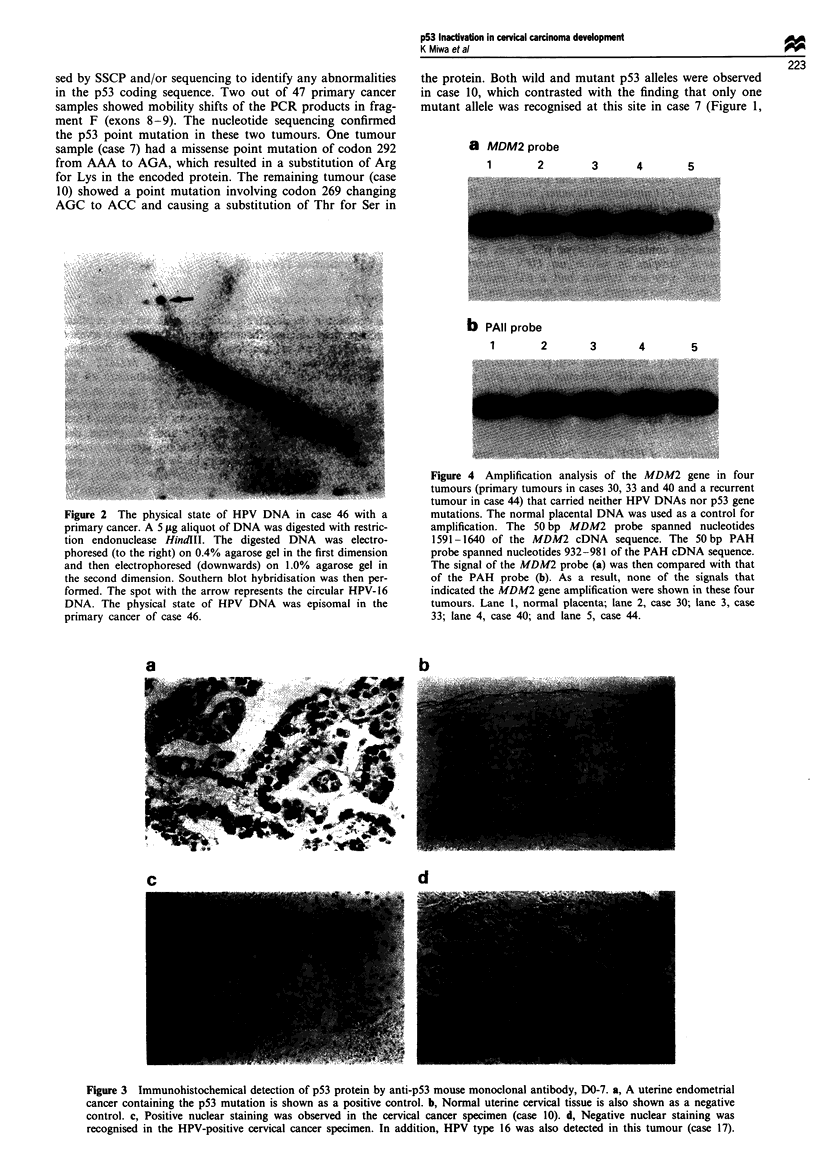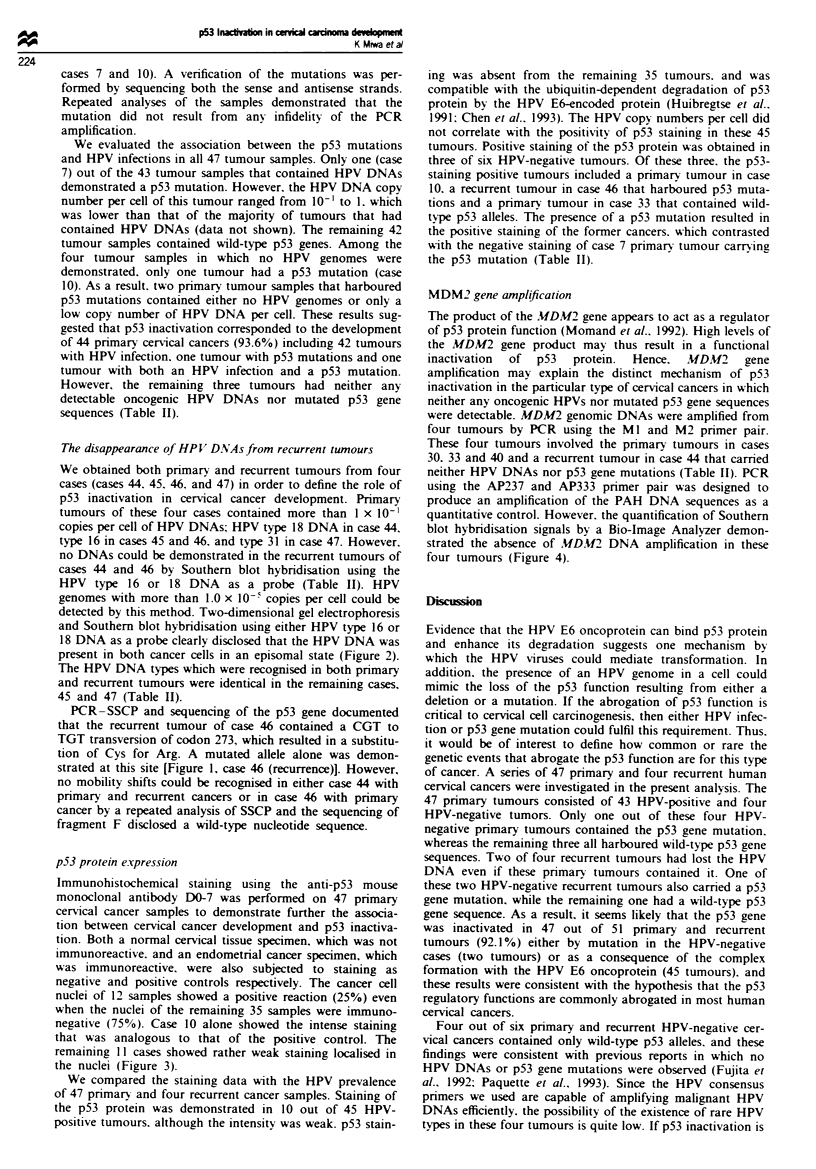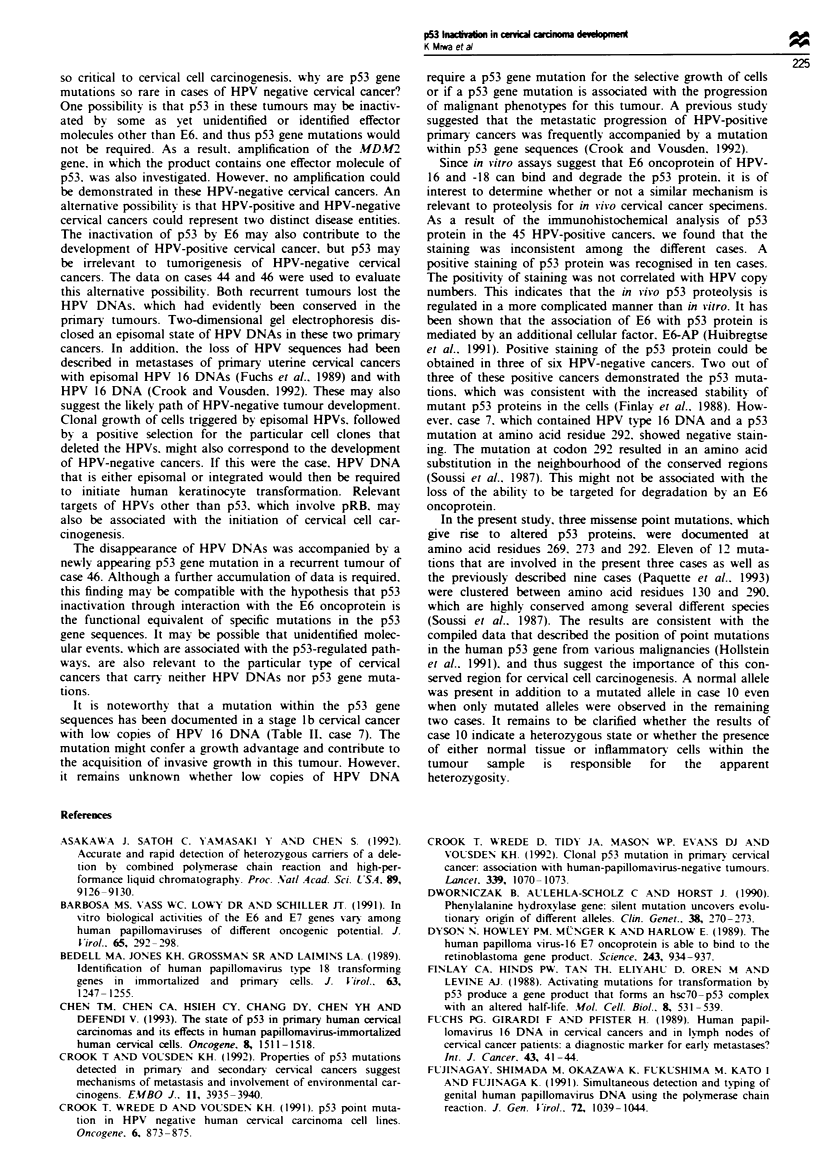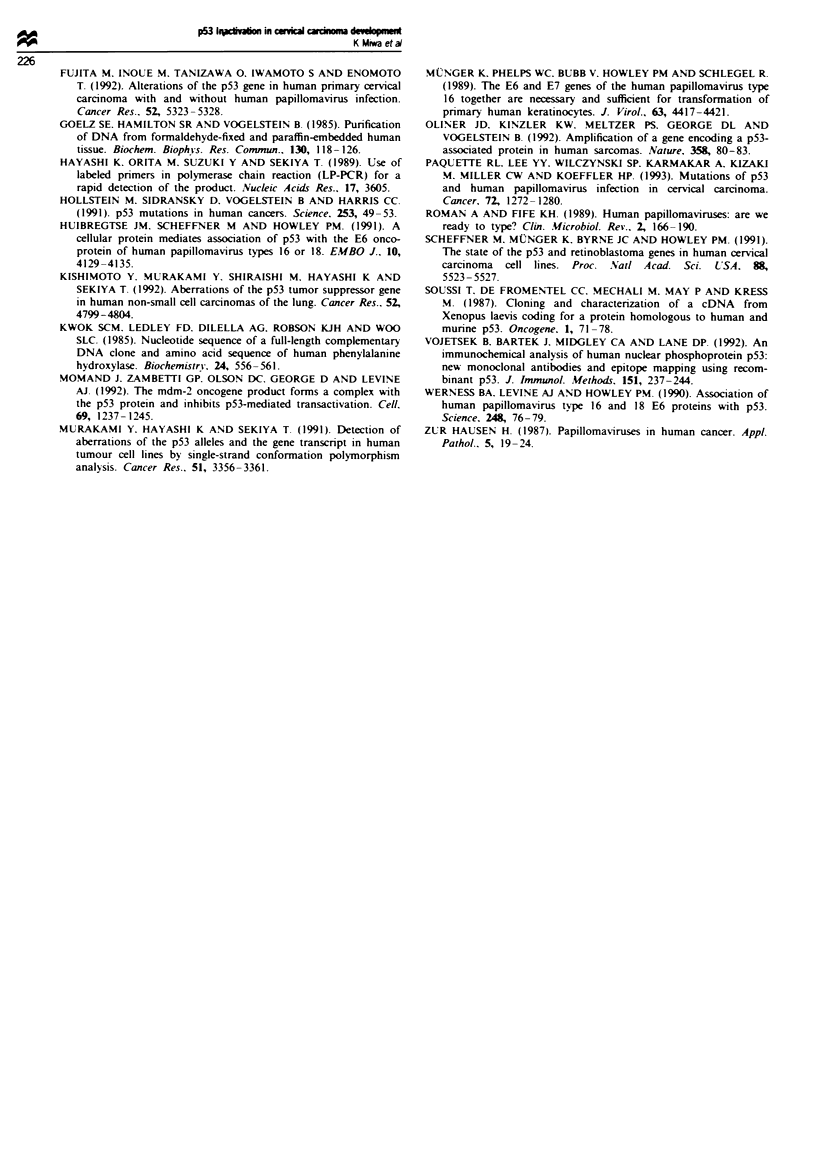# The role of p53 inactivation in human cervical cell carcinoma development.

**DOI:** 10.1038/bjc.1995.47

**Published:** 1995-02

**Authors:** K. Miwa, S. Miyamoto, H. Kato, T. Imamura, M. Nishida, Y. Yoshikawa, Y. Nagata, N. Wake

**Affiliations:** Department of Obstetrics and Gynecology, Faculty of Medicine, Kagoshima University, Japan.

## Abstract

**Images:**


© 1995 Stockton Press All rights reserved 0007-0920/95 $9.00

### The role of p53 inactivation in human cervical cell carcinoma development

K Miwa[1,2], S Miyamoto[2], H Kato[2], T Imamura[2], M Nishida[2], Y Yoshikawa[3], Y Nagata[1] and N Wake[2]

[1]*Department of Obstetrics and Gynecology, Faculty of Medicine, Kagoshima University, 8-35-1, Sakuragaoka, Kagoshima, 890, Japan;* [2]*Departments of Reproductive Physiology and Endocrinology and* [3]*Department of Inspection, Medical Institute of Bioregulation, Kyushu University, 4546, Tsurumihara, Beppu, 874, Japan.*

**Summary** We investigated the association between human papillomavirus (HPV) infection and p53 gene mutation in 47 primary uterine cervical cancers. HPV DNA sequences were present in 43 cancers (91.5%), and one of these cancers contained a p53 gene mutation. In addition, one of the remaining four HPV-negative cancers also contained a p53 gene mutation. As a result, p53 inactivation corresponded to the development of 44 of the primary uterine cervical cancers studied (93.6%). We obtained both primary and recurrent tumours from four cases. In two of these cases, the HPV genomes that were present in an episomal state in the primary tumours were observed to have disappeared in the recurrent tumours. One of these recurrent tumours also contained a p53 gene mutation, which suggested the possibility that p53 inactivation was required in order to maintain the aggressive behaviour in this cancer either by an HPV infection or by a p53 gene mutation. No *MDM2* gene amplification was observed in the tumours that carried neither HPV DNAs nor p53 gene mutations.

**Keywords:** cervical carcinoma; HPV; p53 inactivation; *MDM2* amplification

Uterine cervical cancer is the most common genital malignancy, and a strong association with human papillomaviruses (HPVs) has been suggested (zur Hausen, 1987). More than 65 different types of HPVs have been described. A subgroup of these viruses, including HPV types 16, 18, 31, 33, 52b and 58, has been aetiologically implicated in cervical cell carcinogenesis (Fujinaga *et al.*, 1991) because they are found in a high percentage of these cancers and because benign lesions, with which these viruses are usually associated, are considered to be precursors for malignant progression. Only part of the HPV genome, encoding the E6 and E7 proteins, is consistently retained and expressed in cancer cells. The coordinated expression of E6 and E7 has been shown to contribute to the transformation of rodent cells and the immortalisation of primary human keratinocytes (Bedell *et al.*, 1989; Münger *et al.*, 1989; Barbosa *et al.*, 1991). One important function of the E6 oncoprotein is its ability to form a complex with the cellular p53 protein (Werness *et al.*, 1990), resulting in an ubiquitin-dependent degradation of the latter protein. Likewise, the E7 oncoprotein also forms a complex with the cell-encoded retinoblastoma protein (pRb) (Dyson *et al.*, 1989).

Mutations in the p53 gene are frequently observed in a wide variety of human malignancies (Hollstein *et al.*, 1991). It is thus conceivable that an abrogation of p53 gene functions could be implicated in the aetiology of cancers. Such p53 gene mutations have also been documented in cervical cancers and their cell lines. Only HPV-negative cervical cancers carry p53 mutations (Crook *et al.*, 1992), an observation compatible with the assumption that the abrogation of the p53 gene functions is caused either by binding with the HPV E6-encoded protein or by a mutation associated with cervical cell carcinogenesis (Crook *et al.*, 1991; Scheffner *et al.*, 1991). However, this hypothesis still remains inconclusive because the p53 inactivation may not be obligatory for a few HPV-negative cancers (Fujita *et al.*, 1992). Several possibilities have to be considered in the development of cancers that carry neither the HPV genome nor a p53 gene mutation. First, HPV DNAs or p53 gene mutations could exist but could simply be undetectable by standard methods. Second, mutations could exist in other genes that interact with p53 or downstream of p53 and thus result in an identical physiological defect within the cell. Third, mutations in other genes, which are totally unrelated to p53, could occur in some cancers, resulting in a transformation process that is qualitatively different from HPV-mediated carcinogenesis. Thus, the present study was undertaken to define the molecular mechanism associated with HPV-negative cancer development.

The polymerase chain reaction (PCR) and subsequent restriction enzyme typing are sufficiently sensitive to detect a subgroup of HPVs associated with cervical cancer. Both a sense primer, pU-1M, that is homologous to a region in the E6 open reading frame (ORF) and an antisense primer, pU-2R, that is homologous to a region in the E7 ORF were used for amplification. This method is also useful in identifying potentially new HPV types (Fujinaga *et al.*, 1991). Southern blot hybridisation in conjunction with polymerase chain reaction (PCR) was able to detect $10^{-5}$ to $10^{-6}$ copies of the HPV genome per cell, which was sufficient to determine the presence of HPVs (Roman and Fife, 1989). We thus studied the frequency of HPV infection in 51 cervical cancers that involved both primary and recurrent tumours, and found four primary and two recurrent tumours that were HPV negative. The p53 gene mutation was recognised in 1 of the 45 HPV-positive tumours and in two of six HPV-negative tumours. The HPV genomes that were present as episomes in two primary cancers were observed to have disappeared in the recurrent tumours, and thus suggested the possible route of HPV-negative cancer development.

The disappearance of the HPV genome was followed by the appearance of a new p53 mutation in one of these two recurrent tumours. These findings might thus imply both p53 inactivation in the initiation of cervical cell carcinogenesis and the maintenance of transformed properties even if the frequency of p53 mutation was low in HPV-negative cancers.

The closest analogue to the interaction between E6 and p53 in cervical cancers is provided by the murine double minute 2 (*MDM2*) genes and p53 in sarcomas. An *MDM2* gene product which binds to p53 has been shown to be amplified in a subset of sarcomas (Oliner *et al.*, 1992). If a major effect of *MDM2* gene amplification were inactivation of the p53 protein, then cervical cancers with the *MDM2*

Correspondence: K Miwa
Received 25 May 1994; revised 12 September 1994; accepted 19 September 1994

gene amplification, which are devoid of both HPV DNAs and p53 mutations, would be expected. However, no *MDM2* gene amplification was associated with the four cancers that carried neither HPV DNAs nor p53 gene mutations.

### Materials and methods

### Samples and DNA extraction

Samples for this study were obtained from 51 patients with 47 primary and four recurrent uterine cervical cancers who underwent a biopsy at the Department of Obstetrics and Gynecology, Medical Institute of Bioregulation, Kyushu University. All specimens had been fixed in 10% neutral formalin and embedded in paraffin. The tumours were histologically classified according to the WHO histological typing system. The clinical stage of the disease was based on the International Federation of Gynecology and Obstetrics staging system. Genomic DNA was extracted from formalin-fixed and paraffin-embedded tissue samples using previously described procedures (Goelz *et al.*, 1985).

### Detection and typing of HPV DNA

The detection and typing of HPV DNA were performed using the polymerase chain reaction (PCR) as previously described (Fujinaga *et al.*, 1991). Briefly, the reaction mixture of PCR contained 1.0 μg of DNA, 100 pmol of each consensus primer (pU-1M, 5'-TGTCAAAAACCGTTGTGTCC-3'; pU-2R, 5'-GAGCTGTCGCTTAATTGCTC-3') 200 μM of each dNTP, 50 mM potassium chloride, 10 mM Tris-HCl pH 8.3, 1.5 mM-magnesium chloride, 100 μg ml$^{-1}$ gelatin and 2.5 units of *Taq* DNA polymerase in a total volume of 100 μl. One cycle of PCR consisted of denaturation (94°C for 1 min), primer annealing (55°C for 2 min) and extension (72°C for 2 min) for 30 cycles (Thermo Cycler; Perkin-Elmer Cetus). The PCR product (10 μl) was electrophoresed on 2% agarose gel and stained with ethidium bromide and photographed under UV light. For the typing of HPV DNA, the residual PCR product was digested with restriction enzymes (*Ava*II, *Rsa*I, *Bgl*II and *Acc*I) and the digested products were then analysed on 2% agarose gel. The PCR products in which no HPV DNA was detected by the previous method were then further examined for the presence of HPV-16 or -18 using Southern blot hybridisation. They were electrophoresed on 1.5% agarose gel and denatured in a denaturing solution (1.5 M sodium chloride, 0.5 M sodium hydroxide) and neutralised in a neutralising solution (1.5 M sodium chloride, 0.5 M Tris-HCl pH 7.2, 0.001 M EDTA), and then were transferred to a nylon membrane (Hybond -N$^+$, Amersham). Prehybridisation was carried out in 5 × SSPE, 5 × Denhardt's solution, 0.5% SDS and 100 μg ml$^{-1}$ denatured salmon sperm overnight at 65°C. Hybridisation was done for 12-24 h at 65°C in the same solution as used for prehybridisation with $^{32}$P-labelled probes, using a random-primer method. HPV-16 and -18 probes were synthesised by PCR using DNA derived from SiHa (HPV-16) and HeLa (HPV-18) cells respectively, as described above. The membranes were washed twice in 2 × SSPE, 0.1% SDS, at room temperature for 10 min, once in 1 × SSPE, 0.1% SDS, at 65°C for 15 min, and if necessary once in 0.1% SSPE, 0.1% SDS, at 65°C for 10 min, and then autoradiographed.

### PCR-single-strand conformation polymorhism (SSCP) analysis

PCR-SSCP analysis was performed to detect mutations of the p53 gene as described previously (Hayashi *et al.*, 1989; Murakami *et al.*, 1991; Kishimoto *et al.*, 1992). The primers for the PCR were designed to produce the DNA fragments which covered the entire coding region (from codon 1 to codon 393) of the p53 gene (shown in Table I). Aliquots of 0.1 μg of DNA were subjected to the PCR, using $^{32}$P- end-labelled primers. One PCR cycle consists of denaturation (95°C for 1 min), primer annealing (55°C for 2 min) and extension (72°C for 2 min) for 30 cycles. The PCR product was diluted with a loading solution (95% formamide, 20 mM EDTA, 0.05% xylene cyanol, 0.05% bromphenol blue) and was denatured at 80°C, and then applied to 5% non-denaturing acrylamide gel (acrylamide-methylene-bis acrylamde, 99:1), either with or without 5% glycerol. Electrophoresis was performed at 40 W for 4-7 h while being cooled with a fan. The gel was dried on filter paper and exposed to X-ray film at room temperature for 1-12 h with an intensifying screen.

### Cloning and sequencing

Fragment F from codon 262 to codon 331 of the p53 gene which showed a mobility shift in the PCR-SSCP analysis was sequenced to confirm and localise any point mutations. Since the primers used for the PCR-SSCP analysis had no extraneous nucleotides including any restriction of the enzyme-cut site, new primers, which had extraneous nucleotides including *Eco*RI and *Hind*III sites, where used for PCR in fragment F (F1, 5'-CGAATTCTGAGTAGTGGT-AATCT-3'; F2, 5'-CCCAAGCTTTAGTACCTGAAGGGT-3'). PCR products were purified, digested with *Eco*RI and *Hind*III, and ligated into the *Eco*RI and *Hind*III sites of pUC18 plasmid. Sequencing was performed by the dideoxy sequencing reaction with BcaBEST DNA polymerase (TaKaRa), using fluorescein isothiocyanate (FITC)-labelled primers (FITC primer M4, 5'-CGCCAGGGTTTTCCCAGTCAC-GAC-3'; FITC primer RV-M, 5'-GAGCGGATAACAATT-TCACACAGG-3'; TaKaRa).

**Table I** Primers used for the amplification of the p53 gene

| Amplified DNA fragment | | | | Primer | |
|---|---|---|---|---|---|
| *Name* | *Region* | *Codon* | *Length (bp)* | *Name* | *Sequence* |
| A | Exons 2-3 | 1-32 | 185 | A1 | 5'-TGGAT CCTCT TGCAG CAGCC-3' |
| | | | | A2 | 5'-AACCC TTGTC CTTAC CAGAA-3' |
| B | Exon 4 | 33-125 | 293 | B1 | 5'-ATCTA CAGTC CCCCT TGCCG-3' |
| | | | | B2 | 5'-GCAAC TGACC GTGCA AGTCA-3' |
| C | Exons 5-6 | 126-201 | 325 | C1 | 5'-TTCCT CTTCC TGCAG TACTC-3' |
| | | | | C2 | 5'-GCAAA TTTCC TTCCA CTCGG-3' |
| D | Exons 5-6 | 179-224 | 236 | D1 | 5'-ACCAT GAGCG CTGCT CAGAT-3' |
| | | | | D2 | 5'-AGTTG CAAAC CAGAC CTCAG-3' |
| E | Exon 7 | 225-261 | 139 | E1 | 5'-GTGTT GTCTC CTAGG TTGGC-3' |
| | | | | E2 | 5'-CAAGT GGCTC CTGAC CTGGA-3' |
| F | Exons 8-9 | 262-331 | 330 | F1 | 5'-CCTAT CCTGA GTAGT GGTAA-3' |
| | | | | F2 | 5'-CCAAG ACTTA GTACC TGAAG-3' |
| G | Exon 10 | 332-367 | 139 | G1 | 5'-TGTTG CTGCA GATCC GTGGG-3' |
| | | | | G2 | 5'-GAGGT CACTC ACCTG GAGTG-3' |
| H | Exon 11 | 368-393 | 202 | H1 | 5'-TCTCC TACAG CCACC TGAAG-3' |
| | | | | H2 | 5'-CTGAC GCACA CCTAT TGCAA-3' |

Gel electrophoresis, data collection and analysis were performed using a DSQ-1 DNA sequencer (Shimadzu). A minimum of ten individual clones were sequenced for each specimen. Each sequencing reaction was performed twice, and in each case the presence of the mutation was confirmed.

*Two-dimensional gel electrophoresis and Southern blot hybridisation*

Two-dimensional gel electrophoresis and Southern blot hybridisation, using either HPV-16 or -18 DNA as a probe, were performed to confirm the physical states of HPV DNA in two primary tumours (cases 44 and 46, Table II) in which HPV DNA was present in the primary sample but absent from the recurrent sample. A 5 μg aliquot of each DNA specimen was digested with restriction endonuclease *Hind*III not cleaving within the HPV-16 and -18 genomes. The digested DNA samples were electrophoresed on 0.4% agarose gel at 50 V for 5 h in the first dimension and then electrophoresed on 1.0% agarose gel at 50 V for 5 h in the second dimension. The procedures of denaturing, neutralisation, transferring, prehybridisation, hybridisation and washing were all performed as described above.

**Table II** The status of HPV prevalence, p53 expression and p53 gene

| *Case* | *Stage* | *Histology* | *Site* | *HPV* | *p53 expression* | *p53 gene (codon)* |
|---|---|---|---|---|---|---|
| 1 | Ia | SCC | P | 16 | – | wt |
| 2 | Ia | SCC | P | 16 | – | wt |
| 3 | Ia | SCC | P | 18+58 | – | wt |
| 4 | Ia | SCC | P | 16 | + | wt |
| 5 | Ia | SCC | P | 52b | + | wt |
| 6 | Ib | SCC | P | 16 | – | wt |
| 7 | Ib | SCC | P | 16 | – | AAA→AGA(292) |
| 8 | Ib | SCC | P | 18 | – | wt |
| 9 | Ib | AD | P | 16 | – | wt |
| 10 | Ib | SCC | P | – | ++ | AGC→ACC(269) |
| 11 | Ib | SCC | P | 16 | + | wt |
| 12 | IIb | SCC | P | 16 | – | wt |
| 13 | IIb | AD | P | 16 | – | wt |
| 14 | IIb | SCC | P | 18 | – | wt |
| 15 | IIb | SCC | P | 18 | – | wt |
| 16 | IIb | SCC | P | 16 | + | wt |
| 17 | IIb | SCC | P | 16 | – | wt |
| 18 | IIb | SCC | P | 52b | + | wt |
| 19 | IIIb | SCC | P | 31 | – | wt |
| 20 | IIIb | SCC | P | 58 | – | wt |
| 21 | IIIb | SCC | P | 16 | – | wt |
| 22 | IIIb | SCC | P | 16 | – | wt |
| 23 | IIIb | SCC | P | 18+58 | – | wt |
| 24 | IIIb | SCC | P | 16 | – | wt |
| 25 | IIIb | SCC | P | unknown | + | wt |
| 26 | IIIb | SCC | P | 16 | + | wt |
| 27 | IIIb | SCC | P | 58 | – | wt |
| 28 | IIIb | AD | P | 16 | – | wt |
| 29 | IIIb | SCC | P | 18 | – | wt |
| 30 | IIIb | SCC | P | – | – | wt |
| 31 | IIIb | SCC | P | 31 | – | wt |
| 32 | IIIb | SCC | P | 16 | – | wt |
| 33 | IIIb | SCC | P | – | + | wt |
| 34 | IIIb | SCC | P | 16 | + | wt |
| 35 | IIIb | SCC | P | 16 | + | wt |
| 36 | IIIb | SCC | P | unknown | + | wt |
| 37 | IIIb | SCC | P | 16 | – | wt |
| 38 | IIIb | SCC | P | 16 | – | wt |
| 39 | IIIb | SCC | P | 58 | – | wt |
| 40 | IIIb | AD | P | – | – | wt |
| 41 | IIIb | SCC | P | 52b | – | wt |
| 42 | IIIb | SCC | P | 16 | – | wt |
| 43 | IVa | SCC | P | 16 | – | wt |
| 44 | Ib | SCC | P | 18 | – | wt |
| | Ib | SCC | R | – | – | wt |
| 45 | IIb | SCC | P | 16 | – | wt |
| | IIb | SCC | R | 16 | – | wt |
| 46 | IIIb | SCC | P | 16 | – | wt |
| | IIIb | SCC | R | – | + | GCT→TGT(273) |
| 47 | IVa | AD-SCC | P | 31 | – | wt |
| | IVa | SCC | R | 31 | – | wt |

SCC, squamous cell carcinoma; AD, adenocarcinoma; AD-SCC, adenosquamous cell carcinoma; P, primary; R, recurrence; unknown, type unknown; wt, wild type.

*Immunohistochemistry*

Immunohistochemical detection was performed using the anti-p53 mouse monoclonal antibody DO-7 (NCL-p53-DO-7, Novocastra Laboratories) (Vojetsek *et al.*, 1992). Tumour sections (5 μm) from the paraffin blocks were dewaxed in xylene and passed through alcohol and then washed in phosphate-buffered saline (PBS). Endogenous peroxidase activity was blocked by immersing the sections for 20 min in methanol containing 0.3% hydrogen peroxide.

The sections were incubated with normal goat serum for 20 min at room temperature to reduce the background staining caused by non-specific binding. Primary antibody D0-7, diluted in 1:100 in PBS, was then applied to each section. Bound primary antibody was detected using the streptavidin–biotin–peroxidase system (Dako LSAB kit, Dako) according to the manufacturer's recommendations. A known immunopositive case of human endometrial cancer which demonstrated the p53 mutation was used as a positive control, and normal uterine cervical tissue was used as a negative control. The slides received a methyl green counterstain and were dehydrated in alcohol and xylene before mounting. Only the tumours which exhibited intense nuclear staining throughout the malignant epithelium were categorised as positive cases. The staining intensity was graded as 2+ in more than 50% of the cells, while it was 1+ in less than 50% of the cells.

*PCR and Southern blot hybridisation of* MDM2 *gene*

*MDM2* gene amplification in four samples that carried neither HPV DNAs nor p53 gene mutations was examined by using PCR and Southern blot hybridisation. The primers for PCR of the *MDM2* gene were designed to produce the DNA fragment (201 bp) which spanned nucleotides 1587–1787 of the published *MDM2* cDNA sequence (Oliner *et al.*, 1992) (M1, 5′-GTGGAATCTAGTTTGCCCCTT-3′; M2, 5′-CTAGGGGAAATAAGTTAGCAC-3′). As a reference for the assessment of *MDM2* gene amplification, the primers for PCR of phenylalanine hydroxylase (PAH) gene, which was also located on chromosome 12q, were designed to produce the DNA fragment (213 bp) which spanned exon 7 to part of intron 7 of PAH DNA sequence (Dworniczak *et al.*, 1990) (AP237, 5′-CCCAAACCTCATTCTTGCAGCA-3′; AP333, 5′-CTTGCACTGGTTTCCGCCTC-3′). Two oligonucleotide probes were synthesised by a Cyclone Plus DNA synthesiser (MilliGen Biosearch, Burlington, MA, USA) and were then used for hybridisation. The 50 bp *MDM2* probe spanned nucleotides 1591–1640 of the published *MDM2* cDNA sequence (5′-AATCTAGTTTGCCCCTTAATGCCATTGA-ACCTTGTGTGATTTGTCAAGGT-3′). The 50 bp PAH probe spanned nucleotides 932–981 of the published PAH cDNA sequence (Kwok *et al.*, 1985) (5′-GCACTGGTTTCC-GCCTCCGACCTGTGGCTGGCCTGCTTTCCTCTCGG-GAT-3′). The signal of the *MDM2* probe was then compared with that of the PAH probe. A 50 ng aliquot of DNA was subjected to PCR. One cycle of PCR consisted of denaturation (95°C for 1 min), primer annealing (55°C for 2 min), and extension (72°C for 2 min), for 25 cycles (Asakawa *et al.*, 1992). The PCR products were electrophoresed on 1.5% agarose gel. The procedures of denaturing, neutralisation, transferring, prehybridisation, hybridisation and washing were all performed as described above. The signals were also quantitated on a BAS1000, Bio-imaging Analyzer (Fujix).

### Results

*Pathology and HPV infection in primary cancers*

The histopathological appearance of 47 primary uterine cervical cancers was squamous cell carcinoma in 42 samples

(89.4%), adenocarcinoma in four (8.5%) and adenosquamous cell carcinoma in one (2.1%). We tested the sensitivity of HPV DNA detection by PCR using the pU-1M and pU-2R primer pair. The aliquots corresponding to $1.0 \times 10^{2}$ to $1.0 \times 10^{-5}$ copies per cell of HPV type 16 DNA were subjected to PCR, and then the products were electrophoresed on 2% agarose gel. DNA fragments could be produced by PCR of an aliquot corresponding to $1.0 \times 10^{-1}$ copies per cell. Aliquots in which no HPV type 16 DNA was amplified were further analysed by Southern blot hybridisation using HPV type 16 DNA as a probe. HPV type 16 DNA with more than $1 \times 10^{-5}$ copies per cell could be detected by this method (data not shown), and the findings were similar to previous results (Roman and Fife, 1989; Fujinaga *et al.*, 1991). PCR of genomic DNAs with the pU-1M and pU-2R primer pair and subsequent restriction enzyme analyses, which were able to detect HPV types 16, 18, 31, 33, 52b and 58, as well as unknown types (Fujinaga *et al.*, 1991), showed that 42 out of 47 primary cancers contained HPV DNAs. The remaining five PCR products, in which no HPV DNAs were demonstrated, were further analysed by Southern blot hybridisations using HPV type 16 and 18 DNAs as probes. HPV type 16 DNA was detected in one PCR product, although no types of HPV DNA were recognised in the remaining four products. As a result, the HPV DNA sequences were present in 43 out of 47 primary uterine cancer samples (91.5%), including 25 with HPV type 16 (58.1%, five with type 18 (11.6%), three with type 31 (6.9%), three with type 52b (6.9%) and three with type 58 (6.9%). However, the HPV type was undetermined in two cases (4.6%) while a mixed infection by both HPV type 18 and 58 was also suggested in two cases (4.6%) (Table II).

*The detection of a p53 gene mutation in primary cancers*

Cervical cancer DNAs were subjected to PCR-mediated amplification of exons 2–11, which covered the entire coding region of the p53 gene. The amplified fragments were analy-

**Figure 1** The detection of p53 gene mutations by both PCR–SSCP analysis and sequencing. **a**, PCR–SSCP analysis of the fragment F (exons 8–9) of the p53 gene is shown. Electrophoresis was performed in 5% non-denaturing acrylamide gel containing 5% glycerol. Lane N, normal placenta; lane T, mobility shifts were displayed in three cancers: cases 7 and 10 (primary) and case 46, which demonstrated a recurrent cancer. The arrow indicates the band shift [cases 7, 10, and 46 (recurrence)]. **b**, Nucleotide sequence analysis of fragment F of the p53 gene from the normal placenta. **c**, Nucleotide sequence analysis of fragment F of the p53 gene from uterine cervical cancers [cases 7, 10 and 46 (recurrence)]. An AAA→AGA transversion occurred in codon 292, resulting in Lys→Arg substitution in case 7, an AGC→ACC transversion in codon 269, resulting in a Ser→Thr substitution in case 10 and a CGT→TGT transversion in codon 273, resulting in an Arg→Cys substitution in case 46 (recurrence).

sed by SSCP and/or sequencing to identify any abnormalities in the p53 coding sequence. Two out of 47 primary cancer samples showed mobility shifts of the PCR products in fragment F (exons 8–9). The nucleotide sequencing confirmed the p53 point mutation in these two tumours. One tumour sample (case 7) had a missense point mutation of codon 292 from AAA to AGA, which resulted in a substitution of Arg for Lys in the encoded protein. The remaining tumour (case 10) showed a point mutation involving codon 269 changing AGC to ACC and causing a substitution of Thr for Ser in the protein. Both wild and mutant p53 alleles were observed in case 10, which contrasted with the finding that only one mutant allele was recognised at this site in case 7 (Figure 1,

**Figure 2** The physical state of HPV DNA in case 46 with a primary cancer. A 5 $\mu g$ aliquot of DNA was digested with restriction endonuclease *Hind*III. The digested DNA was electrophoresed (to the right) on 0.4% agarose gel in the first dimension and then electrophoresed (downwards) on 1.0% agarose gel in the second dimension. Southern blot hybridisation was then performed. The spot with the arrow represents the circular HPV-16 DNA. The physical state of HPV DNA was episomal in the primary cancer of case 46.

**Figure 4** Amplification analysis of the *MDM2* gene in four tumours (primary tumours in cases 30, 33 and 40 and a recurrent tumour in case 44) that carried neither HPV DNAs nor p53 gene mutations. The normal placental DNA was used as a control for amplification. The 50 bp *MDM2* probe spanned nucleotides 1591–1640 of the *MDM2* cDNA sequence. The 50 bp PAH probe spanned nucleotides 932–981 of the PAH cDNA sequence. The signal of the *MDM2* probe (**a**) was then compared with that of the PAH probe (**b**). As a result, none of the signals that indicated the *MDM2* gene amplification were shown in these four tumours. Lane 1, normal placenta; lane 2, case 30; lane 3, case 33; lane 4, case 40; and lane 5, case 44.

**Figure 3** Immunohistochemical detection of p53 protein by anti-p53 mouse monoclonal antibody, D0-7. **a**, A uterine endometrial cancer containing the p53 mutation is shown as a positive control. **b**, Normal uterine cervical tissue is also shown as a negative control. **c**, Positive nuclear staining was observed in the cervical cancer specimen (case 10). **d**, Negative nuclear staining was recognised in the HPV-positive cervical cancer specimen. In addition, HPV type 16 was also detected in this tumour (case 17).

cases 7 and 10). A verification of the mutations was performed by sequencing both the sense and antisense strands. Repeated analyses of the samples demonstrated that the mutation did not result from any infidelity of the PCR amplification.

We evaluated the association between the p53 mutations and HPV infections in all 47 tumour samples. Only one (case 7) out of the 43 tumour samples that contained HPV DNAs demonstrated a p53 mutation. However, the HPV DNA copy number per cell of this tumour ranged from $10^{-1}$ to 1, which was lower than that of the majority of tumours that had contained HPV DNAs (data not shown). The remaining 42 tumour samples contained wild-type p53 genes. Among the four tumour samples in which no HPV genomes were demonstrated, only one tumour had a p53 mutation (case 10). As a result, two primary tumour samples that harboured p53 mutations contained either no HPV genomes or only a low copy number of HPV DNA per cell. These results suggested that p53 inactivation corresponded to the development of 44 primary cervical cancers (93.6%) including 42 tumours with HPV infection, one tumour with p53 mutations and one tumour with both an HPV infection and a p53 mutation. However, the remaining three tumours had neither any detectable oncogenic HPV DNAs nor mutated p53 gene sequences (Table II).

### *The disappearance of HPV DNAs from recurrent tumours*

We obtained both primary and recurrent tumours from four cases (cases 44, 45, 46, and 47) in order to define the role of p53 inactivation in cervical cancer development. Primary tumours of these four cases contained more than $1 \times 10^{-1}$ copies per cell of HPV DNAs; HPV type 18 DNA in case 44, type 16 in cases 45 and 46, and type 31 in case 47. However, no DNAs could be demonstrated in the recurrent tumours of cases 44 and 46 by Southern blot hybridisation using the HPV type 16 or 18 DNA as a probe (Table II). HPV genomes with more than $1.0 \times 10^{-5}$ copies per cell could be detected by this method. Two-dimensional gel electrophoresis and Southern blot hybridisation using either HPV type 16 or 18 DNA as a probe clearly disclosed that the HPV DNA was present in both cancer cells in an episomal state (Figure 2). The HPV DNA types which were recognised in both primary and recurrent tumours were identical in the remaining cases, 45 and 47 (Table II).

PCR-SSCP and sequencing of the p53 gene documented that the recurrent tumour of case 46 contained a CGT to TGT transversion of codon 273, which resulted in a substitution of Cys for Arg. A mutated allele alone was demonstrated at this site [Figure 1, case 46 (recurrence)]. However, no mobility shifts could be recognised in either case 44 with primary and recurrent cancers or in case 46 with primary cancer by a repeated analysis of SSCP and the sequencing of fragment F disclosed a wild-type nucleotide sequence.

### *p53 protein expression*

Immunohistochemical staining using the anti-p53 mouse monoclonal antibody D0-7 was performed on 47 primary cervical cancer samples to demonstrate further the association between cervical cancer development and p53 inactivation. Both a normal cervical tissue specimen, which was not immunoreactive, and an endometrial cancer specimen, which was immunoreactive, were also subjected to staining as negative and positive controls respectively. The cancer cell nuclei of 12 samples showed a positive reaction (25%) even when the nuclei of the remaining 35 samples were immunonegative (75%). Case 10 alone showed the intense staining that was analogous to that of the positive control. The remaining 11 cases showed rather weak staining localised in the nuclei (Figure 3).

We compared the staining data with the HPV prevalence of 47 primary and four recurrent cancer samples. Staining of the p53 protein was demonstrated in 10 out of 45 HPV-positive tumours, although the intensity was weak. p53 staining was absent from the remaining 35 tumours, and was compatible with the ubiquitin-dependent degradation of p53 protein by the HPV E6-encoded protein (Huibregtse *et al*., 1991; Chen *et al*., 1993). The HPV copy numbers per cell did not correlate with the positivity of p53 staining in these 45 tumours. Positive staining of the p53 protein was obtained in three of six HPV-negative tumours. Of these three, the p53-staining positive tumours included a primary tumour in case 10, a recurrent tumour in case 46 that harboured p53 mutations and a primary tumour in case 33 that contained wild-type p53 alleles. The presence of a p53 mutation resulted in the positive staining of the former cancers, which contrasted with the negative staining of case 7 primary tumour carrying the p53 mutation (Table II).

### *MDM2 gene amplification*

The product of the *MDM2* gene appears to act as a regulator of p53 protein function (Momand *et al*., 1992). High levels of the *MDM2* gene product may thus result in a functional inactivation of p53 protein. Hence, *MDM2* gene amplification may explain the distinct mechanism of p53 inactivation in the particular type of cervical cancers in which neither any oncogenic HPVs nor mutated p53 gene sequences were detectable. *MDM2* genomic DNAs were amplified from four tumours by PCR using the M1 and M2 primer pair. These four tumours involved the primary tumours in cases 30, 33 and 40 and a recurrent tumour in case 44 that carried neither HPV DNAs nor p53 gene mutations (Table II). PCR using the AP237 and AP333 primer pair was designed to produce an amplification of the PAH DNA sequences as a quantitative control. However, the quantification of Southern blot hybridisation signals by a Bio-Image Analyzer demonstrated the absence of *MDM2* DNA amplification in these four tumours (Figure 4).

### Discussion

Evidence that the HPV E6 oncoprotein can bind p53 protein and enhance its degradation suggests one mechanism by which the HPV viruses could mediate transformation. In addition, the presence of an HPV genome in a cell could mimic the loss of the p53 function resulting from either a deletion or a mutation. If the abrogation of p53 function is critical to cervical cell carcinogenesis, then either HPV infection or p53 gene mutation could fulfil this requirement. Thus, it would be of interest to define how common or rare the genetic events that abrogate the p53 function are for this type of cancer. A series of 47 primary and four recurrent human cervical cancers were investigated in the present analysis. The 47 primary tumours consisted of 43 HPV-positive and four HPV-negative tumors. Only one out of these four HPV-negative primary tumours contained the p53 gene mutation, whereas the remaining three all harboured wild-type p53 gene sequences. Two of four recurrent tumours had lost the HPV DNA even if these primary tumours contained it. One of these two HPV-negative recurrent tumours also carried a p53 gene mutation, while the remaining one had a wild-type p53 gene sequence. As a result, it seems likely that the p53 gene was inactivated in 47 out of 51 primary and recurrent tumours (92.1%) either by mutation in the HPV-negative cases (two tumours) or as a consequence of the complex formation with the HPV E6 oncoprotein (45 tumours), and these results were consistent with the hypothesis that the p53 regulatory functions are commonly abrogated in most human cervical cancers.

Four out of six primary and recurrent HPV-negative cervical cancers contained only wild-type p53 alleles, and these findings were consistent with previous reports in which no HPV DNAs or p53 gene mutations were observed (Fujita *et al*., 1992; Paquette *et al*., 1993). Since the HPV consensus primers we used are capable of amplifying malignant HPV DNAs efficiently, the possibility of the existence of rare HPV types in these four tumours is quite low. If p53 inactivation is so critical to cervical cell carcinogenesis, why are p53 gene mutations so rare in cases of HPV negative cervical cancer? One possibility is that p53 in these tumours may be inactivated by some as yet unidentified or identified effector molecules other than E6, and thus p53 gene mutations would not be required. As a result, amplification of the *MDM2* gene, in which the product contains one effector molecule of p53, was also investigated. However, no amplification could be demonstrated in these HPV-negative cervical cancers. An alternative possibility is that HPV-positive and HPV-negative cervical cancers could represent two distinct disease entities. The inactivation of p53 by E6 may also contribute to the development of HPV-positive cervical cancer, but p53 may be irrelevant to tumorigenesis of HPV-negative cervical cancers. The data on cases 44 and 46 were used to evaluate this alternative possibility. Both recurrent tumours lost the HPV DNAs, which had evidently been conserved in the primary tumours. Two-dimensional gel electrophoresis disclosed an episomal state of HPV DNAs in these two primary cancers. In addition, the loss of HPV sequences had been described in metastases of primary uterine cervical cancers with episomal HPV 16 DNAs (Fuchs *et al.*, 1989) and with HPV 16 DNA (Crook and Vousden, 1992). These may also suggest the likely path of HPV-negative tumour development. Clonal growth of cells triggered by episomal HPVs, followed by a positive selection for the particular cell clones that deleted the HPVs, might also correspond to the development of HPV-negative cancers. If this were the case, HPV DNA that is either episomal or integrated would then be required to initiate human keratinocyte transformation. Relevant targets of HPVs other than p53, which involve pRB, may also be associated with the initiation of cervical cell carcinogenesis.

The disappearance of HPV DNAs was accompanied by a newly appearing p53 gene mutation in a recurrent tumour of case 46. Although a further accumulation of data is required, this finding may be compatible with the hypothesis that p53 inactivation through interaction with the E6 oncoprotein is the functional equivalent of specific mutations in the p53 gene sequences. It may be possible that unidentified molecular events, which are associated with the p53-regulated pathways, are also relevant to the particular type of cervical cancers that carry neither HPV DNAs nor p53 gene mutations.

It is noteworthy that a mutation within the p53 gene sequences has been documented in a stage Ib cervical cancer with low copies of HPV 16 DNA (Table II, case 7). The mutation might confer a growth advantage and contribute to the acquisition of invasive growth in this tumour. However, it remains unknown whether low copies of HPV DNA require a p53 gene mutation for the selective growth of cells or if a p53 gene mutation is associated with the progression of malignant phenotypes for this tumour. A previous study suggested that the metastatic progression of HPV-positive primary cancers was frequently accompanied by a mutation within p53 gene sequences (Crook and Vousden, 1992).

Since *in vitro* assays suggest that E6 oncoprotein of HPV-16 and -18 can bind and degrade the p53 protein, it is of interest to determine whether or not a similar mechanism is relevant to proteolysis for *in vivo* cervical cancer specimens. As a result of the immunohistochemical analysis of p53 protein in the 45 HPV-positive cancers, we found that the staining was inconsistent among the different cases. A positive staining of p53 protein was recognised in ten cases. The positivity of staining was not correlated with HPV copy numbers. This indicates that the *in vivo* p53 proteolysis is regulated in a more complicated manner than *in vitro*. It has been shown that the association of E6 with p53 protein is mediated by an additional cellular factor, E6-AP (Huibregtse *et al.*, 1991). Positive staining of the p53 protein could be obtained in three of six HPV-negative cancers. Two out of three of these positive cancers demonstrated the p53 mutations, which was consistent with the increased stability of mutant p53 proteins in the cells (Finlay *et al.*, 1988). However, case 7, which contained HPV type 16 DNA and a p53 mutation at amino acid residue 292, showed negative staining. The mutation at codon 292 resulted in an amino acid substitution in the neighbourhood of the conserved regions (Soussi *et al.*, 1987). This might not be associated with the loss of the ability to be targeted for degradation by an E6 oncoprotein.

In the present study, three missense point mutations, which give rise to altered p53 proteins, were documented at amino acid residues 269, 273 and 292. Eleven of 12 mutations that are involved in the present three cases as well as the previously described nine cases (Paquette *et al.*, 1993) were clustered between amino acid residues 130 and 290, which are highly conserved among several different species (Soussi *et al.*, 1987). The results are consistent with the compiled data that described the position of point mutations in the human p53 gene from various malignancies (Hollstein *et al.*, 1991), and thus suggest the importance of this conserved region for cervical cell carcinogenesis. A normal allele was present in addition to a mutated allele in case 10 even when only mutated alleles were observed in the remaining two cases. It remains to be clarified whether the results of case 10 indicate a heterozygous state or whether the presence of either normal tissue or inflammatory cells within the tumour sample is responsible for the apparent heterozygosity.

### References

ASAKAWA J, SATOH C, YAMASAKI Y AND CHEN S. (1992). Accurate and rapid detection of heterozygous carriers of a deletion by combined polymerase chain reaction and high-performance liquid chromatography. *Proc. Natl Acad. Sci. USA*, **89**, 9126–9130.

BARBOSA MS, VASS WC, LOWY DR AND SCHILLER JT. (1991). In vitro biological activities of the E6 and E7 genes vary among human papillomaviruses of different oncogenic potential. *J. Virol.*, **65**, 292–298.

BEDELL MA, JONES KH, GROSSMAN SR AND LAIMINS LA. (1989). Identification of human papillomavirus type 18 transforming genes in immortalized and primary cells. *J. Virol.*, **63**, 1247–1255.

CHEN TM, CHEN CA, HSIEH CY, CHANG DY, CHEN YH AND DEFENDI V. (1993). The state of p53 in primary human cervical carcinomas and its effects in human papillomavirus-immortalized human cervical cells. *Oncogene*, **8**, 1511–1518.

CROOK T AND VOUSDEN KH. (1992). Properties of p53 mutations detected in primary and secondary cervical cancers suggest mechanisms of metastasis and involvement of environmental carcinogens. *EMBO J.*, **11**, 3935–3940.

CROOK T, WREDE D AND VOUSDEN KH. (1991). p53 point mutation in HPV negative human cervical carcinoma cell lines. *Oncogene*, **6**, 873–875.

CROOK T, WREDE D, TIDY JA, MASON WP, EVANS DJ AND VOUSDEN KH. (1992). Clonal p53 mutation in primary cervical cancer: association with human-papillomavirus-negative tumours. *Lancet*, **339**, 1070–1073.

DWORNICZAK B, AULEHLA-SCHOLZ C AND HORST J. (1990). Phenylalanine hydroxylase gene: silent mutation uncovers evolutionary origin of different alleles. *Clin. Genet.*, **38**, 270–273.

DYSON N, HOWLEY PM, MÜNGER K AND HARLOW E. (1989). The human papilloma virus-16 E7 oncoprotein is able to bind to the retinoblastoma gene product. *Science*, **243**, 934–937.

FINLAY CA, HINDS PW, TAN TH, ELIYAHU D, OREN M AND LEVINE AJ. (1988). Activating mutations for transformation by p53 produce a gene product that forms an hsc70–p53 complex with an altered half-life. *Mol. Cell. Biol.*, **8**, 531–539.

FUCHS PG, GIRARDI F AND PFISTER H. (1989). Human papillomavirus 16 DNA in cervical cancers and in lymph nodes of cervical cancer patients: a diagnostic marker for early metastases? *Int. J. Cancer*, **43**, 41–44.

FUJINAGA Y, SHIMADA M, OKAZAWA K, FUKUSHIMA M, KATO I AND FUJINAGA K. (1991). Simultaneous detection and typing of genital human papillomavirus DNA using the polymerase chain reaction. *J. Gen. Virol.*, **72**, 1039–1044.

FUJITA M, INOUE M, TANIZAWA O, IWAMOTO S AND ENOMOTO T. (1992). Alterations of the p53 gene in human primary cervical carcinoma with and without human papillomavirus infection. *Cancer Res.*, **52**, 5323–5328.

GOELZ SE, HAMILTON SR AND VOGELSTEIN B. (1985). Purification of DNA from formaldehyde-fixed and paraffin-embedded human tissue. *Biochem. Biophys. Res. Commun.*, **130**, 118–126.

HAYASHI K, ORITA M, SUZUKI Y AND SEKIYA T. (1989). Use of labeled primers in polymerase chain reaction (LP-PCR) for a rapid detection of the product. *Nucleic Acids Res.*, **17**, 3605.

HOLLSTEIN M, SIDRANSKY D, VOGELSTEIN B AND HARRIS CC. (1991). p53 mutations in human cancers. *Science*, **253**, 49–53.

HUIBREGTSE JM, SCHEFFNER M AND HOWLEY PM. (1991). A cellular protein mediates association of p53 with the E6 oncoprotein of human papillomavirus types 16 or 18. *EMBO J.*, **10**, 4129–4135.

KISHIMOTO Y, MURAKAMI Y, SHIRAISHI M, HAYASHI K AND SEKIYA T. (1992). Aberrations of the p53 tumor suppressor gene in human non-small cell carcinomas of the lung. *Cancer Res.*, **52**, 4799–4804.

KWOK SCM, LEDLEY FD, DILELLA AG, ROBSON KJH AND WOO SLC. (1985). Nucleotide sequence of a full-length complementary DNA clone and amino acid sequence of human phenylalanine hydroxylase. *Biochemistry*, **24**, 556–561.

MOMAND J, ZAMBETTI GP, OLSON DC, GEORGE D AND LEVINE AJ. (1992). The mdm-2 oncogene product forms a complex with the p53 protein and inhibits p53-mediated transactivation. *Cell*, **69**, 1237–1245.

MURAKAMI Y, HAYASHI K AND SEKIYA T. (1991). Detection of aberrations of the p53 alleles and the gene transcript in human tumour cell lines by single-strand conformation polymorphism analysis. *Cancer Res.*, **51**, 3356–3361.

MÜNGER K, PHELPS WC, BUBB V, HOWLEY PM AND SCHLEGEL R. (1989). The E6 and E7 genes of the human papillomavirus type 16 together are necessary and sufficient for transformation of primary human keratinocytes. *J. Virol.*, **63**, 4417–4421.

OLINER JD, KINZLER KW, MELTZER PS, GEORGE DL AND VOGELSTEIN B. (1992). Amplification of a gene encoding a p53-associated protein in human sarcomas. *Nature*, **358**, 80–83.

PAQUETTE RL, LEE YY, WILCZYNSKI SP, KARMAKAR A, KIZAKI M, MILLER CW AND KOEFFLER HP. (1993). Mutations of p53 and human papillomavirus infection in cervical carcinoma. *Cancer*, **72**, 1272–1280.

ROMAN A AND FIFE KH. (1989). Human papillomaviruses: are we ready to type? *Clin. Microbiol. Rev.*, **2**, 166–190.

SCHEFFNER M, MÜNGER K, BYRNE JC AND HOWLEY PM. (1991). The state of the p53 and retinoblastoma genes in human cervical carcinoma cell lines. *Proc. Natl Acad. Sci. USA*, **88**, 5523–5527.

SOUSSI T, DE FROMENTEL CC, MECHALI M, MAY P AND KRESS M. (1987). Cloning and characterization of a cDNA from Xenopus laevis coding for a protein homologous to human and murine p53. *Oncogene*, **1**, 71–78.

VOJETSEK B, BARTEK J, MIDGLEY CA AND LANE DP. (1992). An immunochemical analysis of human nuclear phosphoprotein p53: new monoclonal antibodies and epitope mapping using recombinant p53. *J. Immunol. Methods*, **151**, 237–244.

WERNESS BA, LEVINE AJ AND HOWLEY PM. (1990). Association of human papillomavirus type 16 and 18 E6 proteins with p53. *Science*, **248**, 76–79.

ZUR HAUSEN H. (1987). Papillomaviruses in human cancer. *Appl. Pathol.*, **5**, 19–24.